# Seed Dispersal by Ants in Three Early-Flowering Plants

**DOI:** 10.3390/insects13040386

**Published:** 2022-04-14

**Authors:** Pavol Prokop, Jana Fančovičová, Zuzana Hlúšková

**Affiliations:** 1 Department of Environmental Ecology and Landscape Management, Faculty of Natural Sciences, Comenius University, Ilkovičova 6, 842 15 Bratislava, Slovakia; 2 Institute of Zoology, Slovak Academy of Sciences, Dúbravská cesta 9, 845 06 Bratislava, Slovakia; 3 Department of Biology, Faculty of Education, Trnava University, 918 43 Trnava, Slovakia; jana.fancovicova@truni.sk; 4 Elementary School Janka Palu 2, 914 41 Nemšová, Slovakia; z.hluskova1@gmail.com

**Keywords:** elaiosome removal, myrmecochory, snowdrop

## Abstract

**Simple Summary:**

Myrmecochory is seed dispersal of numerous plant species mediated by ants. We investigate ant–plant interactions under field conditions across two study sites in Central Europe. Three obligatory myrmecocohrous plants are chosen for the experiments: snowdrop *Galanthus nivalis*, hollow root *Corydalis cava* and European wild ginger *Asarum europaeum*. We experimentally alter diaspore morphology and record seed removal rates across five treatments: elaiosomes without seeds, diaspore without elaiosome, 1/2 elaiosome + diaspore, 1/2 diaspore + elaiosome and control. Elaiosomes of European wild ginger constitute about 30% of diaspore weight, elaiosomes of snowdrop constitute 13% and elaiosomes of hollow root constitute only 7.5%. Diaspore/elaiosome removal rates are highest in European wild ginger (34%), followed by hollow root (26%) and snowdrop (10%). Only two ants interact with diaspores, the acorn ant *Temnothorax crassispinus* and the red ant *Myrmica ruginodis*. Ants respond to elaiosome/seed ratio by removing elaiosomes without diaspores most frequently, followed by 1/2 diaspore + elaiosome, control, diaspores without elaiosomes and 1/2 elaiosome with diaspore. Plants do not effectively manipulate ant behavior and no dispersal benefits from interactions with ants are observed.

**Abstract:**

Interactions between ants and plants vary from being occasionally beneficial to neutral and negative. Ant-mediated dispersal of obligatory myrmecochorous plants is considered mutualistic interaction, providing benefits to plants in terms of seed dispersal. Ants are rewarded by providing elaiosome, sugar, lipid and protein-rich appendages attached to seeds (diaspores). We experimentally examine rates of diaspore removal rates among three species of plants (snowdrop *Galanthus nivalis*, hollow root *Corydalis cava* and European wild ginger *Asarum europaeum*) under field conditions in two study sites in Central Europe. Diaspore morphology is altered by manipulating both elaiosome and seed size. The small-sized acorn ant *Temnothorax crassispinus* interacts with the snowdrop and hollow root and the moderately-sized red ant *Myrmica ruginodis* interacts with European wild ginger. Experimental manipulation with elaiosomes yields largely non-significant results. Diaspore removal rates are generally low (snowdrop 10%, hollow root 26%, European wild ginger 34%) probably due to the small size of ants relative to heavy diaspores. Many ants are observed to consume elaiosomes in situ (cheating). We conclude that ant–plant relationships in this case are not mutualistic but rather neutral/slightly negative, because the plants do not obtain any apparent benefits from their interactions with ants.

## 1. Introduction

Competition is a strong driver of mutualism [1], because mutualism evolved in environments where competition for food resources and predation pressures were more intense [2,3,4]. Interspecific interactions for (probably) mutualistic commodities often vary from occasionally beneficial to neutral or negative [5], because these commodities are costly for providers [6,7] (Pringle 2016, Harcombe et al., 2018).

Myrmecochory is considered a mutualistic interaction between ants and seeds [8], observed in more than 11,000 angiosperm species [9] (Lengyel et al., 2010). Myrmecochorous plants produce diaspores with sugar, lipid and protein-rich appendages (elaiosomes) attached to them [10,11] (Hughes et al., 1994, Rico-Gray, & Oliveira, 2007). Elaiosomes serve as rewards for ants and their presence facilitates diaspore removal and its transfer to ant nests where elaiosomes are typically consumed, while the seed remains intact [11,12]. To support reciprocal benefits between plants and ants, research showed support for protection against seed predators, distance dispersal from maternal plants and reduction in intraspecific competition, directed dispersal to nutrient-enriched microsites in ant nests and an increase in ant colony fitness as a result of feeding on elaiosomes (e.g., [13,14,15,16,17,18,19], for the review see [20,21]).

Although the benefits of ants for myrmecochorous plants are obvious (e.g., [9,11,20]), the outcomes of interactions between ants and diaspores are not always clear (e.g., [22,23,24]). First, seed-dispersing ants are not strictly tied to the presence of myrmecochorous plants [15,25] and experimental removal of myrmechorous plants in a 13-year-long experiment showed no effect of diaspore absence on the ants [5]. Second, most ant species prefer arthropod prey over seeds [26,27,28], suggesting that carrying diaspores is less beneficial than feeding on common prey. Third, several plants deceive ants by exploiting their preferences for false elaiosomes with a low concentration of rewarding nutrients [29,30], supporting the idea that ants are susceptible to manipulations by plants [5,27,31].

Seedling survival is positively correlated with seed size [32], and elaiosome size positively correlates with rates of diaspore removal by ants [33,34]; thus, plants need to solve the trade-off between investment in seed mass and elaiosome mass [33,35]. Since ants are removing diaspores together with elaiosomes, but carrying loads decrease their locomotory speed [36], evolution should favor individuals who are able to discriminate diaspores with reactively greater elaiosomes, which function as better rewards to ants.

Ant preference for relative elaiosome mass within species is a crucial ecological trait influencing the evolution of seed dispersal by ants [33]. Although the morphology of elaiosomes was investigated by measuring their size relative to the seed (e.g., [34]), experimental altering of seed mass relative to elaiosome mass is very scarce (experiments on Australian ants [37]). We investigate diaspore removal rates in three myrmecochorous plant species in field experiments with two species of ants common in Central Europe. We specifically examine the hypothesis that ants prefer relatively larger elaiosomes over relatively smaller elaiosomes within each plant species.

## 2. Materials and Methods

### 2.1. Study Species

Three indigenous myrmecochorous early flowering plant species with different diaspore/elaiosome sizes occurring in Slovak forests were used for the experiments: hollow root *Corydalis cava* (L.) Schweigg. et Körte, snowdrop *Galanthus nivalis* (L.) and European wild ginger *Asarum europaeum* (L.).

### 2.2. Study Area

The experiment took place in areas where the study species naturally occur and when their diaspores are ripe. Hollow root and snowdrop were studied between 22 April and 9 May and 30 May and 19 June 2017 in Vlčkovský háj (48.28639° N, 17.64955° E), respectively. European wild ginger was studied in Tlstá Hora (49.02319° N, 18.13099° E) between 27 July and 10 August 2017. Vlčkovský háj is a small foodplain forest with dominant trees such as oak (*Quercus* spp.), common ash (*Fraxinus excelsior*) and maple (*Acer* spp.). Other early flowering understorey herbs, apart from the study species, are two-leaf squill (*Scilla bifolia*) and the yellow anemone (*Anemone ranunculoides*). The locality on Tlstá Hora is characterized by a deciduous forest covered predominantly by European beech (*Fagus sylvatica*) and similar trees compared to Vlčkovský háj. In contrast to the previous locality, European wild ginger is the only dominant understorey herb.

### 2.3. Experimental Procedure

**Figure 1:**
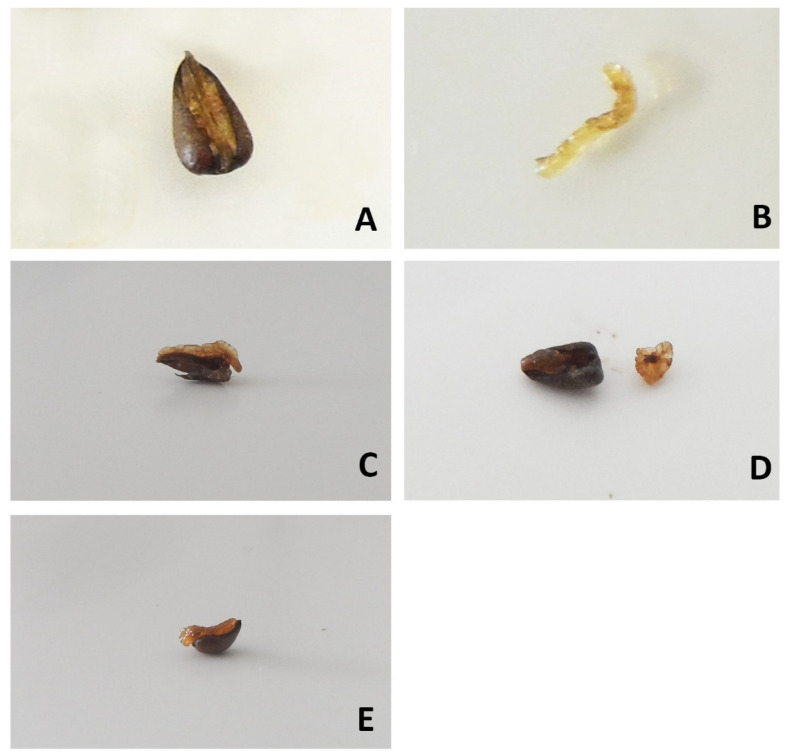
Examples of experimental treatments in the European wild ginger. Diaspore without elaiosome (**A**), elaiosome only (**B**), 1/2 diaspore + elaiosome (**C**), 1/2 elaiosome + diaspore (**D**) and control (**E**).

Three to five days before the experiment started, the ripe diaspores of all three species were collected from the study sites and frozen at −20 °C according to a recommendation by Englický and Šerá [38]. One day before the experiment, randomly selected diaspores were divided into five treatments: treatment with removed elaiosomes without seeds (Elaiosome only), diaspores with elaiosome removed (Diaspore without elaiosome), diaspores with half of the elaiosome removed (1/2 Elaiosome + Diaspore), elaiosomes with half of the diaspore removed (1/2 Diaspore + Elaiosome) and intact diaspores (Control) (Figure 1). Half of all the diaspores/elaiosomes of the snowdrop and hollow root and all diaspores/elaiosomes of European wild ginger were weighed on an analytical balance to the nearest 0.0001 g. The ratio of elaiosome wet mass to diaspore wet mass was calculated for all three species of plants. Since elaiosomes were extremely small, which could negatively influence the accuracy of our measurements, we always weighed six diaspores/elaiosomes together. Diaspores/elaiosomes were finally refrigerated at 4 °C and used for the field experiment on the subsequent day.

For each species, we placed 10 × 10 cm index cards at 1 m intervals in a line along the forest floor, with 6 diaspores/elaiosomes for each card. Each card contained diaspores/elaiosomes of one treatment following Gunther and Lanza [39]. We used replications of our experiment to address possible confounding effects of local factors, such as proximity to the nearest colony, number of aboveground foragers on a particular day, prior food saturation of the local habitat, etc. The order of treatments was randomized. We recorded (1) the arrival time of the first ant, (2) the maximum number of ants, (3) the time when the first diaspore/elaiosome was removed by ants out of the index card and (4) the numbers of diaspores remaining per card at 5-min intervals for a period of 90 min. For statistical analyses, maximum first ant arrival time and the time when the first diaspore/elaiosome was removed was 90 min. Several specimens of ants were collected from index cards after the experiment finished, stored in alcohol, and determined in the laboratory according to Seifert [40].

The time of year when the experiments were conducted coincides with the time that these species ripen. The experiment was replicated four times for each species and all records were made by two of us (PP and JF). Ten cards per each treatment were used for the experimental day for hollow root and snowdrop (50 trials per day and 200 trials per all 4 experimental days). Five cards per treatment (25 trials per day and 100 trials per all 4 experimental days) were used for the European ginger. ZH was trained by PP and JF before the experiment to ensure that all data will be collected identically across species.

All experiments were conducted in shady places of the forest during windless and rainless days with minimum temperature >15 °C between 8 am and 4 pm to standardize experimental conditions.

### 2.4. Statistical Analyses

Data were checked for normality with Shapiro–Wilk test and parametric or non-parametric statistical tests were used accordingly. Differences between absolute (overall differences between diaspores/elaiosomes across treatments) and relative weights (% of elaiosome mass from total diaspore mass calculated for control treatment) of diaspores/elaiosomes were calculated with ANOVA and subsequent comparisons between means were made with the Fisher pos-hoc test. The ant’s arrival time and elaiosome/diaspore removal were analyzed with Kruskal–Wallis ANOVA. The maximum number of ants and the total number of diaspores/elaiosomes that remained in index cards (removal rates) was analyzed with General Linear Model (GLM) with Poisson distribution where treatment was defined as categorical predictor. Partial correlations between variables were controlled for the effect of plant species, treatment, and day of the experiment. All statistical tests were performed by SPSS ver. 26.

## 3. Results

### 3.1. Ants Participating in Seed Dispersal

Only one ant species, the acorn ant *Temnothorax crassispinus* (Karawajew, 1926), was determined on diaspores/elaiosomes of snowdrop and hollow root. The red ant *Myrmica ruginodis* (Nylander, 1846) was the only species found on diaspores/elaiosomes of the European wild ginger.

### 3.2. Absolute and Relative Differences of Diaspore/Elaiosome Weight among Plants

There were significant differences in mean weight on intact (control) diaspores (ANOVA, *F*_2,57_ = 35.45, *p* < 0.001). Diaspores of snowdrop were heavier than diaspores of hollow root (post-hoc *p* = 0.013) and European wild ginger (post-hoc *p* < 0.001). Diaspores of hollow root were heavier than diaspores of European wild ginger (post-hoc *p* < 0.001). The absolute weights of elaiosomes were different between species (*F*_2,57_ = 45.35, *p* < 0.001). Elaiosomes of European wild ginger were the heaviest, followed by snowdrop and hollow root (post-hoc *p* ≤ 0.001). The relative weight of elaiosomes also differed between species (*F*_2,57_ = 194.52, *p* < 0.001). Elaiosomes of European wild ginger constituted (mean ± SE) 29.91 ± 0.83% of the diaspore weight, while elaiosomes of snowdrop 13.47 ± 0.83% and elaiosomes of hollow root only 7.60 ± 0.83% of total diaspore weight (post-hoc tests, all *p* < 0.001).

### 3.3. Effects of Treatment on Diaspore/Elaiosome Weight

Unsurprisingly, intact diaspores (control group) were always the heaviest, and elaiosomes always showed the lowest weight in comparison with other groups (all post-hoc *p* < 0.001, Table 1). 1/2 elaiosome + diaspore treatments were heavier than diaspores without elaiosomes (post-hoc tests, hollow root and European wild ginger, *p* < 0.001), but this difference was not statistically significant for snowdrop (post-hoc test, *p* = 0.084). Furthermore, 1/2 diaspore + elaiosome seeds were lighter than diaspore without elaiosome (post-hoc tests, snowdrop and hollow root, *p* < 0.001), but this difference was not statistically significant for European wild ginger (post-hoc test, *p* = 0.148).

### 3.4. First Ant Arrival Time

**Figure 2:**
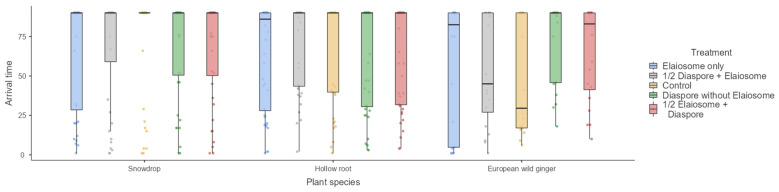
First ant arrival time (min) observed on diaspores/elaiosomes among three plant species. Box plots represent medians, 25th and 75th percentiles, minimum and maximum values.

The effect of the treatment on the first ant arrival time was not significant (Kruskal–Wallis H_4_ = 1.80, 1.88 and 6.56, *p* = 0.77, 0.76 and 0.15) for the snowdrop (interacted with *T. crassispinus*), hollow root (interacted with *T. crassispinus*) and European wild ginger (interacted with *M. ruginodis*), respectively (Figure 2).

### 3.5. Maximum Number of Ants

**Figure 3:**
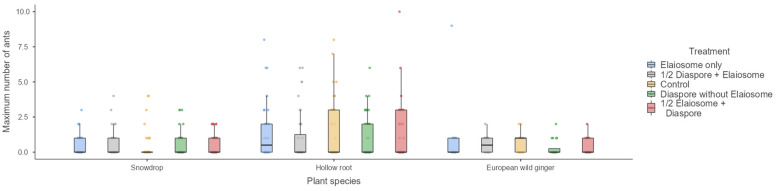
Maximum number of ants observed on diaspores/elaiosomes among three plant species. Box plots represent medians, 25th and 75th percentiles, minimum and maximum values.

Maximum number of ants on the index card was not significantly influenced by treatment (GLM, Wald’s χ^2^ = 0.65, 2.96 and 5.55, df = 1,4, *p* = 0.96, 0.57 and 0.24) in the snowdrop (interacted with *T. crassispinus*), hollow root (interacted with *T. crassispinus*) and European wild ginger (interacted with *M. ruginodis*), respectively (Figure 3).

### 3.6. Diaspore/Elaiosome Removal

**Figure 4:**
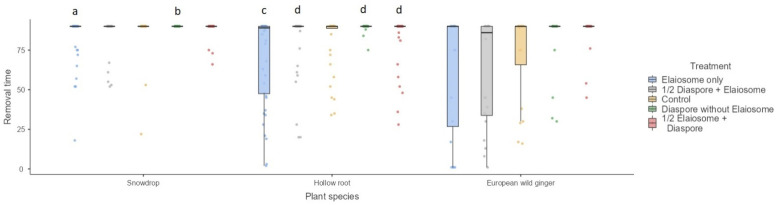
Mean first diaspore/elaiosome removal time (min) among three plant species. Box plots represent medians, 25th and 75th percentiles, minimum and maximum values. The letters above the bars denote significant differences according to pair-wise comparisons (a vs. b, *p* < 0.01, c vs. d, *p* < 0.05).

The removal time was significantly influenced by treatment (Kruskal–Wallis H_4_ = 13.33, 21.8 and 10.61) in the snowdrop (interacted with *T. crassispinus,* *p* = 0.01), hollow root (interacted with *T. crassispinus,* *p* < 0.001) and European wild ginger (interacted with *M. ruginodis,* *p* = 0.03), respectively (Figure 4). With respect to European wild ginger, differences between treatments were not significant after Bonferroni corrections.

**Figure 5:**
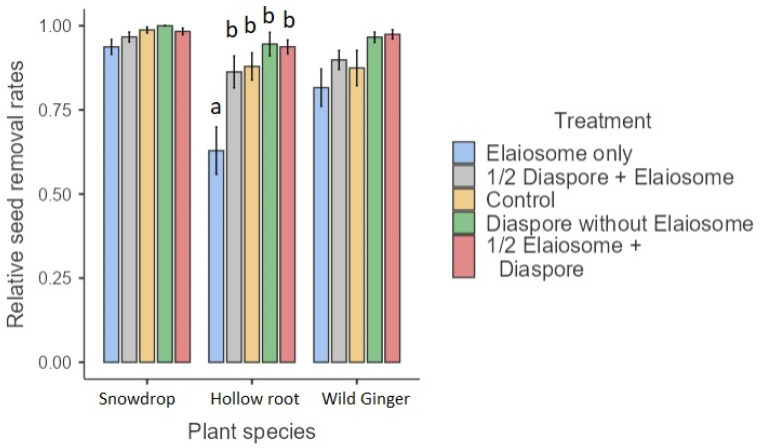
Diaspore/elaiosome removal rates among plant species and treatments. The letters above the bars denote significant differences (a vs. b, *p* ≤ 0.01).

The occurrence of snowdrop diaspores/elaiosomes removal was apparently less frequent (19/200, 10%, interacted with *T. crassispinus*) than removal of European wild ginger (34/100, 34%, interacted with *M. ruginodis*) or hollow root (51/200, 26%, interacted with *T. crassispinus*). Ants that did not transport diaspores/elaiosomes were frequently observed to feed elaiosomes in situ. The effect of treatment on diaspore/elaiosome removal rates was significant only for the hollow root (interacted with *T. crassispinus*) (GLM, Wald’s χ^2^ = 18.92, df = 4, *p* = 0.001) but not for snowdrop (interacted with *T. crassispinus*) and European wild ginger (interacted with *M. ruginodis*) (GLM, Wald’s χ^2^ = 0.57 and 2.30, df = 4, *p* = 0.97 and 0.98, respectively). Elaiosomes without diaspores were removed most frequently, followed by ½ diaspore + elaiosome and control (Figure 5).

The highest diaspores/elaiosomes removal rates were found for hollow root (Mean = 0.86, SE = 0.03), followed by European ginger (Mean = 0.91, SE = 0.03) and snowdrop (Mean = 0.98, SE = 0.03).

### 3.7. Associations between Ants and Diaspore/Elaiosome Displacement

After controlling for the effect of plant species, treatment and day, we found that when the first ant arrived to diaspores/elaiosomes quickly, the total number of observed ants was higher, diaspore/elaiosome transport was faster and diaspores/elaiosomes were displaced more quickly than when the arrival time was longer (Table 2). The more ants that were observed near diaspores/elaiosomes, the quicker the transport of the first diaspore/elaiosome occurred and the fewer diaspores/elaiosome remained on the index card. If the transport of the first diaspore/elaiosome was delayed, the diaspore/elaiosome removal was less intense.

## 4. Discussion

This study aimed to experimentally investigate the role of relative elaiosome and seed size in ant attraction among three early flowering understorey herbs under natural conditions. By manipulating both elaiosome and seed size, we found that the diaspores of these species attract two ant species and that diaspore removal rates are influenced by relative elaiosome/seed mass which supports the importance of diaspore morphology for ant attraction [29]. As far as we are aware, this is also the first attempt to investigate myrmecochory in the snowdrop, *G. nivalis*.

Both the acorn ant *T. crassispinus* [34,41,42] and the red ant *M. ruginodis* [18,42] are common in Central Europe. These ants are known to carry and disperse diaspores of myrmecochorous plants, although their role in seed dispersal is far less investigated in comparison with other ant species [18,41]. Many acorn ants are small-sized ants (body length often within 2.3–3.4 mm [43]) and thus efficient for the dispersal of small-seeded myrmecochorous plants with soft elaiosomes, such as *Moehringia* spp. or *Chelidonium majus* [41]. Workers of the red ant *M. ruginodis* are of moderate size (4.0–6.0 mm [44,45]) and prefer diaspores of moderate size, such as *Viola riviniana*, *C. cava*, *Scilla bifolia*, *Hepatica nobilis, Ulex* spp. or *Carex pilulifera* [18,34,46,47]). Our observations show that the acorn ants *T. crassispinus* interact with the snowdrop and the red ant *M. ruginodis* interacts with the European wild ginger in natural conditions.

Ant activity on index cards with diaspores/elaiosomes was influenced by several variables. Median values for the European wild ginger show that diaspores/elaiosomes were occupied by ants sooner than the remaining species. With respect to possible differences in chemical attraction, elaiosomes of hollow root contain more oleic acid than the European ginger [45], one of the most important ant-attractant components of myrmecochorous plants [29,48]. Unfortunately, we are not aware of data on the chemical composition of elaiosomes of the snowdrop, so further comparisons of this species are not possible.

Diaspore/elaiosome removal rates were significantly influenced by treatment. Elaiosomes of the hollow root were removed at higher rates compared with other treatments, supporting the conventional view that the presence of elaiosome is a crucial factor that favors diaspore dispersal by ants (e.g., [20,21,45]). Elaiosomes of the snowdrop were largely ignored, however, suggesting that they can contain chemicals attracting other ant species and/or that their large weight is unsuitable for carrying by the acorn ant.

Ant responses on experimentally treated diaspores suggests that they are able to discriminate between food items with the highest (strong preference for the elaiosome treatment followed by 1/2 diaspore with elaiosome treatment and control) and the lowest reward (lowest preferences for 1/2 elaiosome + diaspore and diaspore without elaiosome treatment) [37,49] and that relative elaiosome weight influences ant behavior [33,34,35]. This suggests that the studied plants do not deceive these two ant species [29,30]. We suggest that the low susceptibility of the acorn ant *T. crassispinus* and the red ant *M. ruginodis* to possible manipulation by myrmecochorous plants [5,27,31] studied here can be explained by ants’ low interest in diaspore removal. The majority of diaspores were not removed at least during a 90 min period and diaspores of snowdrop have been largely ignored by ants. This suggests that the acorn ant and the red ant are possibly not the primary dispersers of the three plant species in the study area and their contribution to plant dispersal is marginal. On the one hand, these results are not surprising given that laboratory experiments showed that the closely related *Temnothorax nylanderi* ant rejected diaspores of hollow root [45]. The red ant *M. ruginodis* in the same experiment, however, showed a preference for the European wild ginger [45], which was not fully confirmed in our field experiment. Similarly, Gorb and Gorb [50], studying the diaspore removal rates of the European ginger (among others), found evidence of diaspore removal by *Formica polyctena* and questioned the significant role of diaspore dispersal of this species by the red ant *Myrmica rubra*. Possibly, certain gastropods, rather than ants, may play a crucial role in diaspore dispersal of European wild ginger [51].

Although ants are a major selective force toward seed dispersal by arthropods (e.g., [11]), we observed low rates of diaspore dispersal in three myrmecochorous early flowering plants interacting with two ant species of small and moderate size. These results are particularly surprising for the snowdrop, because this plant is at least partly self-compatible [52] and selfing plants are expected to invest more in seed dispersal relative to plants with a high outcrossing rate [53].

## 5. Conclusions

In conclusion, ant–plant interactions in two localities of Central Europe could be considered as neutral or slightly negative in terms of seed dispersal, because the diaspore removal rates were very low. These results are in all probability influenced by ants of small and moderate sizes which are not effective for transporting diaspores of myrmecochorous plants with large diaspores. We suggest that these ant species encountered different plants due to different ecological conditions which favor the presence of one or the other ant species rather than by a different preference that each species has. Interestingly, however, the acorn ants *T. crassispinus* prefer elaiosomes without seeds in a hollow root more than seeds with elaiosomes, suggesting that they can discriminate between food items with the highest and lowest reward. These discrimination abilities mean that plants do not manipulate ant behavior effectively and the plant benefit from seed dispersal by these two ant species is low. Future research should consider possible seasonal differences of myrmecochory, because certain ant species may stop the removal of diaspores over time [28] and differences in microsites with different (and possibly larger) ant species.

## Figures and Tables

**Table 1:** Descriptive comparison of diaspores/elaiosomes among five treatments. Mean represents the mean wet weight of six diaspores/elaiosomes (g). Mean and standard error (SE) are provided.

	*G. nivalis*		*C. cava*		*A. europaeum*		*n*
	Mean	SE	Mean	SE	Mean	SE	
Elaiosome only	0.010	0.003	0.005	0.001	0.013	0.0008	20
Diaspore without Elaiosome	0.065	0.003	0.061	0.001	0.029	0.0008	20
1/2 elaiosome + diaspore	0.073	0.003	0.07	0.001	0.039	0.0008	20
1/2 diaspore + elaiosome	0.046	0.003	0.033	0.001	0.028	0.0008	20
Control	0.084	0.003	0.075	0.001	0.057	0.0008	20
ANOVA *F*_4,95_ =	86.96, *p* < 0.001		601.80, *p* < 0.001		439.41, *p* < 0.001		-

**Table 2:** Partial correlations controlled for the effect of plant species, treatment and day between measured variables (*n* = 500). All correlations are significant at *p* < 0.001.

	Maximum Number of Ants	Transport of First Diaspore	Seed Remnant
First ant arrival time	−0.66	0.54	0.43
Maximum number of ants	-	−0.37	−0.45
Transport of first diaspore		-	0.71

## Data Availability

Data are available from the corresponding author upon reasonable request.
